# Routine detection and management of neurocognitive impairment in HIV-positive patients in a UK centre

**DOI:** 10.1177/0956462412472452

**Published:** 2013-03

**Authors:** L J Haddow, A Accoroni, J D Cartledge, H Manji, P Benn, R J C Gilson

**Affiliations:** * Centre for Sexual Health and HIV Research, Research Department of Infection and Population Health, University College London; † Camden Provider Services, Central and North West London NHS Foundation Trust; ‡ University College London Hospitals NHS Foundation Trust, London, UK

**Keywords:** HIV infection, AIDS dementia complex, HIV-associated neurocognitive disorder, asymptomatic neurocognitive impairment, HIV-associated dementia, cross-sectional studies, prevalence

## Abstract

We estimated the burden of HIV-associated neurocognitive disorders (HAND) in a UK clinic. From a random sample, and referrals to specialist services over one year (neurology, clinical psychology, hospital admissions), we determined whether patients were diagnosed with HIV-associated dementia (HAD) and whether they reported symptoms suggesting neurocognitive impairment (NCI). In the first sample, 2/150 (prevalence 1.3%; 95% confidence interval [CI] 0.2–4.7%) had documented HAD. Eleven patients (7.3%; CI 3.7–12.7%) reported recent symptoms suggesting NCI; most of these individuals were diagnosed with a psychiatric or substance-use disorder. Among specialist referrals with symptoms suggesting NCI, 11 were diagnosed with HAD from a clinic population of 3129 individuals (annual incidence 0.4%; CI 0.2–0.6%). No patients with mildly symptomatic or asymptomatic HAND were identified in either sample, suggesting that such patients remain undetected in current clinical practice. Evidence-based screening for HAND in HIV clinics may be needed.

## INTRODUCTION

The most recent British HIV Association (http://www.bhiva.org/TreatmentofHIV1_2012.aspx) and European AIDS Clinical Society (EACS) (http://www.europeanaidsclinicalsociety.org) guidelines for the antiretroviral treatment of HIV-positive adults now support the treatment of patients with symptomatic neurocognitive impairment (NCI) – that is, both minor neurocognitive disorder (MND) and HIV-associated dementia (HAD) – regardless of CD4 count. Only the latter guidelines discuss screening for HIV-associated neurocognitive disorders (HAND) in routine HIV care, recommending a three-question symptom questionnaire^1^ at HIV diagnosis, prior to starting antiretroviral therapy (ART) and at two-year intervals. It is difficult to determine the clinical impact of this new emphasis in the guidelines, partly because of a lack of knowledge of the clinical importance of mild-to-moderate HAND, low levels of detection of such disorders, high rates of cognitive symptoms in those with normal cognitive function and the absence of a strategy for screening and testing in most clinics. We aimed to estimate the current burden of NCI detected in routine HIV clinical practice in a large UK clinic.

## METHODS

We conducted two retrospective cross-sectional surveys. In the first, a sample of 150 individuals was randomly selected from a list of 3129 patients who attended the HIV clinic in the first half of both 2009 and 2010. We reviewed notes in April 2011 to determine whether patients had ever been diagnosed with HAD, the equivalent diagnoses of AIDS dementia complex (ADC) or HIV encephalopathy (HIVE) or milder HAND (MND), and whether they had reported difficulties with cognitive function in the 12-month period preceding the most recent attendance. The diagnoses of HAD, according to two definitions,^2,3^ and AIDS dementia complex^4^ purport to address the same syndrome and have been shown to give similar diagnostic yield.^5^ The sample size gave 90% power to detect a 2.5% or greater prevalence of the outcome. As well as identifying clinicians’ contemporaneous observations of possible NCI, documentation of incidental symptoms was interpreted by researchers during the notes review process. Patients were fully evaluated as indicated by clinical need. However, not all symptomatic patients underwent sufficient assessment of neuropsychological function and activities of daily living to meet current diagnostic criteria for HAND.^2^ Therefore, the diagnostic label of MND may not necessarily have applied in this context. As there was no routine screening for HAND during the data collection period, asymptomatic neurocognitive impairment (ANI) was not recorded in the notes. Other data collected included age, gender, current and nadir CD4 + count, viral load (VL), ART history, alcohol or drug abuse, relevant medical history and neurological and psychiatric co-morbidity.

In the second survey, we reviewed the notes of all referrals to an HIV neurology clinic, referrals with suspected NCI to a specialist clinical psychology service for HIV and sexual health-related problems, and admissions to an HIV inpatient unit with symptoms of chronic cognitive impairment during a similar 12-month period (August 2009 to July 2010). There were no specific criteria for referral to these services, other than the opinion of the patient's HIV outpatient doctor or (in the case of inpatient admissions) the admitting clinician. For each patient, we recorded whether a diagnosis of HAD or a related disorder had been made, as well as additional data as above.

## RESULTS

In the first, random sample (*n* = 150), median age was 43 years (interquartile range [IQR] 38–49 years), 125 (83%) were men, ethnicity was white (*n* = 98, 65%), black African (*n* = 20, 13%) or other (*n* = 32, 21%), route of infection was sex between men (*n* = 106, 71%), heterosexual sex (*n* = 36, 24%), intravenous drug use (*n* = 2, 1.3%), vertical (*n* = 1, 0.7%) or not known (*n* = 5, 3.3%), current CD4+ count was median 540 cells/μL (IQR 400–720 cells/μL), nadir CD4+ was median 195 cells/μL (IQR 130–280 cells/μL) and 129 patients (86%) were taking ART, of whom 123 (95%) had VL <50 copies/mL. These characteristics were representative of the entire clinic population during the same period. Median time since diagnosis was 8.8 years (IQR 5.3–14.5 years) and median duration of ART was 6.6 years (IQR 3.3–11.1 years). Relevant co-morbidity included depression (*n* = 41, 27%), schizophrenia (*n* = 1, 0.7%), significant substance misuse (*n* = 14, 9%; including 8 individuals with heavy alcohol use and 1 each using heroin, alcohol and heroin in combination, *γ*-hydroxybutyric acid [GHB], crack cocaine, anabolic steroids and cannabis), hepatitis C (*n* = 10, 7%), and previous cerebral toxoplasmosis (*n* = 2, 1.3%).

**Figure 1:**
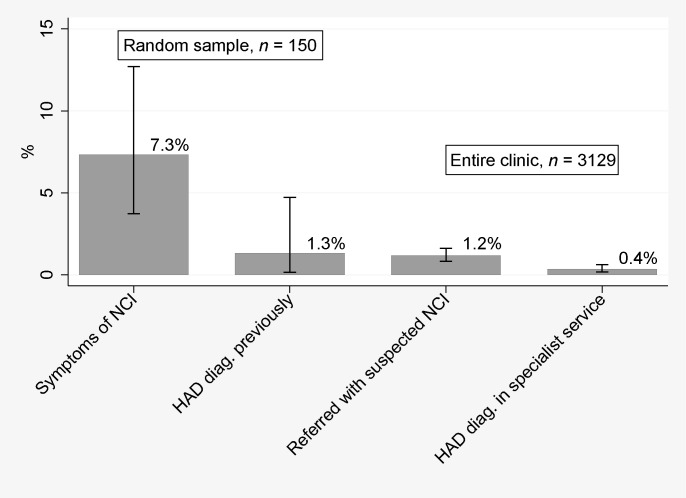
Prevalence estimates with 95% confidence intervals of main reported diagnoses and complaints of interest. HAD, HIV-associated dementia; NCI, neurocognitive impairment

Of these 150 patients, two (1.3%; 95% confidence interval [CI] 0.2–4.7%) had documented HAD (Figure 1), and no patients had been diagnosed with MND or ANI. Eleven patients (7.3%; 95% CI 3.7–12.7%), including the two with HAD, had symptoms suggesting NCI within the last year of attendance. Of these, six (55%) had significant depressive symptoms, two (18%) were attributed to efavirenz and one (9%) had possible NCI that was confounded by depressive and psychological problems when assessed neuropsychologically after the study period.

In the second survey, there were 22 referrals to the HIV neurology clinic, six referrals to clinical psychology and 14 admissions to the HIV inpatient unit with chronic cognitive difficulties, with or without focal neurological symptoms, during a one-year period. One of the clinical psychology patients and four of the hospitalized patients were also seen in neurology clinic, and none were in the random sample of *n* = 150 (above). Chronic cognitive symptoms reported by patients referred to the neurology clinic were memory problems (*n* = 11), global cognitive impairment (*n* = 9), impaired concentration (*n* = 3) and word-finding difficulties (*n* = 2), while associated problems included motor signs and symptoms (*n* = 5), mood disturbance (*n* = 3), altered behaviour (*n* = 2), balance/gait disturbance (*n* = 2) and headache (*n* = 1). Of the five clinical psychology patients not seen by neurology clinic, all complained of memory impairment and two complained of difficulties with attention/concentration. The ward admissions not seen by neurology clinic presented primarily with global cognitive decline (*n* = 10), behavioural disturbance (*n* = 3) or a systemic illness to which chronic cognitive impairment was a secondary problem (*n* = 1).

Thus there were 37 patients with suspected NCI requiring specialist assessment and management from a clinic population of approximately 3129 individuals (overall risk 1.2%; 95% CI 0.8–1.6%) in a one-year period (Figure 1). A final diagnosis of HAD or HIVE was made in six neurology referrals, five hospital admissions and no clinical psychology referrals, totalling 11 new cases in 3129 patients (0.4%; 95% CI 0.2–0.6%) in one year. Common alternative diagnoses were anxiety and depression (12 of 37 patients, 32%) and new or past opportunistic infections (OIs) (5; 14%).

## DISCUSSION

We have estimated a 1.3% prevalence of HAD in a small but representative sample of patients currently attending a large HIV clinic in the UK. Additionally, 7% of patients complained of symptoms consistent with NCI during a one-year period. Also in a one-year period, 1.2% of the clinic population was referred to specialist services for the investigation and management of chronic cognitive symptoms. Most of these symptoms were not attributed to HAND, and the approximate annual incidence of HAD detected through these referrals to specialist services was 0.4%. Our estimates are in keeping with the rates of HAD in recent studies using diverse diagnostic criteria in different study populations in Europe and the USA.^1–2,6–14^ However, the near-absence of mild or moderate HAND detected in routine care is striking when compared with recent prevalence estimates of 10% and 28% for MND, 17% for mild cognitive/motor disorder and 28% and 42% for ANI.^1,2,11^ The UK may have different rates of HAND from other regions due its demographic and clinical characteristics. The HIV-positive population is ageing, with around a fifth of those accessing care aged 50 and above, has over 80% ART coverage with over 90% of those achieving VL <50 copies/mL, and is culturally and ethnically diverse, and 30% of newly diagnosed patients have CD4+ counts of <200 cells/μL at HIV diagnosis.^15–17^ There remains a need for accurate UK and European estimates of the prevalence of all grades of HAND, based on large, unbiased samples and complete assessment according to current diagnostic criteria.^2^

We consider these estimates to reflect recent routine clinical practice and *ad hoc* recording of patients’ symptoms, rather than being a complete representation of the burden of cognitive impairment in HIV-positive individuals. Despite these limitations, our findings have implications for clinical practice. It appears that most patients with MND and ANI will remain undetected, while assessment of patients reporting symptoms consistent with NCI will frequently detect patients with anxiety, depression, OIs and drug and alcohol problems. In the light of our findings, we have set up a multidisciplinary clinic and referral pathway aiming to assess such patients, and will be evaluating the use of the three-symptom questionnaire, as advocated by EACS, in routine clinical practice. It is important to detect patients with MND because the rate of further cognitive and functional decline in these patients, although poorly quantified in the literature, may be between 16% and 52% over six months to two years.^2,5,18,19^ Also, a study conducted in Italy from 1996 to 2004 in a cohort with 41% prevalence of intravenous drug use and 45% hepatitis C co-infection found a 1.4-fold higher risk of virological failure in those who were neurocognitively impaired at baseline, and a 2.9-fold higher rate of death in those with NCI and virological failure compared with unimpaired patients.^20^ We need further evidence on the accuracy of screening tests for ANI and MND, the natural history and appropriate management of patients with mild-to-moderate HAND and the benefits of a screening strategy in various clinical settings.

## Notes

**Funding:** LH was supported by the Medical Research Council UK (Clinical Research Training Fellowship award number G0700530).
